# From Joseph Richardson

**Published:** 1882-10

**Authors:** Joseph Richardson


DR. TAFT.

*Dear Sir*:—Since writing the article which appears in the present number of the REGISTER, I find, in the July, (1882) number of the *American Journal of the Medical Sciences*, the following under the caption, "Storage and Utilization of Phosphates in Pregnancy."

The theory, it will be observed, is in harmony with the claim of the paper, namely that, aside from exceptional cases of tissue-starvation as to the phosphates, there is, ordinarily, a sufficiency of the lime-salts during pregnancy for all the contingent needs of both mother and child.

"In a recent number of the *Union Medicale*, Dr. Delattre discusses a phenomenon of early pregnancy which he considers has not hitherto received the attention which, both on physiological and therapeutical grounds, it deserves. He refers to the almost complete disappearance of the phosphates from the urine. These salts, he says, are, except the small proportion as yet required by the development of the fœtus, either stored up in the maternal bones, which increase in weight and density, or, occasionally, deposited on their surface in the form of osteophytes, which have long been looked upon as errors of nutrition. In the later months, when the fœtal bones are growing and ossifying rapidly, these reserves are drawn on, and the osteophytes, if present, disappear. The absorption is not complete at the time when the child is born, but goes on during the normal time of lactation, supplying phosphates to the milk. Such is the course of events in the case of a healthy and well-nourished woman."

Terre Haute, Ind. JOSEPH RICHARDSON.

